# How Are We Preparing Australian and Aotearoa New Zealand Teachers to Be Health Promotors? Examining Physical Activity, Sleep and Sun Safety in Initial Teacher Education

**DOI:** 10.1002/hpja.70022

**Published:** 2025-03-04

**Authors:** Joseph J. Scott, Alexandra P. Metse, Bronwen M. McNoe, Sally Blane, Sharyn Chin Fat, Justine Osborne, Nicky Muir

**Affiliations:** ^1^ School of Education and Tertiary Access The University of the Sunshine Coast Sippy Downs Australia; ^2^ School of Education Edith Cowan University Perth Australia; ^3^ School of Health The University of the Sunshine Coast Sippy Downs Australia; ^4^ School of Psychology University of Newcastle Callaghan Australia; ^5^ Department of Preventive and Social Medicine University of Otago Dunedin New Zealand; ^6^ Cancer Council Western Australia Subiaco Australia; ^7^ Cancer Council Queensland Fortitude Valley Australia; ^8^ Cancer Council Victoria East Melbourne Australia

**Keywords:** health behaviours, physical activity, prevention, sleep, sun safety, teacher education

## Abstract

**Issue Addressed:**

While physical activity, sleep and sun safety (PASS) have been identified as important modifiable health behaviours and schools and teachers have been identified as vital for health promotion and primary prevention; little is known about how initial teacher education programs across Australia and New Zealand (NZ) are preparing future teachers to deliver PASS‐related curriculum. This study investigated teacher educators' insights on their programs and their graduate's preparedness to plan and teach PASS education.

**Methods:**

Teacher educators (*n* = 98) from Australia and NZ completed a 30‐item electronic survey. Quantitative tests were used to explore differences in the data.

**Results:**

Consistently, time spent on physical activity far outweighed sun safety and sleep with many programs having little or no sleep or sun safety content. Of concern, many indicated they did not agree, or know if their graduates were confident to plan and teach physical activity (28%), sun safety (42%) or sleep (75%) lessons, nor were they aware of the related guidelines, health benefits and risks.

**Conclusions:**

Findings reveal significant variance in what is being offered in Australian and NZ initial teacher education programs. Findings highlight potential gaps in graduate's knowledge of various health behaviours and confidence to plan and teach related content and their preparedness for health promotion.

**So What?:**

Findings highlight a need to include more targeted health promotion education in initial teacher education in Australia and NZ to enable teachers to deliver consistent health promotion messages when they enter school settings to properly support young people's health needs.

## 1 Introduction

Physical activity (PA), sleep and sun safety have all been identified as modifiable lifestyle behaviours in young people to improve health outcomes and reduce the risk of chronic disease, including cancer in later life [1, 2, 3]. Physical inactivity is now the fourth leading risk factor associated with mortality globally [1]. In Australia, less than one in 10 (8.9%) young people meet the national PA guidelines with COVID‐19 exacerbating the downward trend [4, 5, 6]. In Aotearoa New Zealand (NZ), the 2022 PA report card data indicates that less than half (47%) of adolescents (aged 15–18) and only three in five (62%) children (aged 5–14 years) meet recommended PA guidelines [7]. The link between PA, optimal sleep and improvements in health and well‐being is now well established [8]. Poor sleep is also increasingly recognised as a significant risk factor for preventable morbidity and mortality, and is prevalent in young people in Australia with 27% of 12–13 years. olds and 52% of 16–17 years. olds not meeting sleep guidelines on school nights [9]. In NZ, only 7 in 10 adults (69.1%) and almost 4 in 5 (78.1%) children are reported to be meeting the recommended amount of sleep, with disproportionately lower rates reported in disadvantaged groups [10]. While the promotion of these behaviours remains vitally important for the maintenance of young people's health, PA often occurs outdoors, so the management of ultraviolet radiation exposure and associated health impacts is an important consideration when promoting this health behaviour. Despite decades of education and the fact that skin cancer is noted as Australasia's most preventable cancer [11], Australia and NZ have the highest rates of skin cancer in the world [12]. There is consensus that sun exposure in the early years of life significantly increases the likelihood of developing skin cancer during adulthood, and for this reason, young people remain a particularly important group for primary prevention [13].

It is estimated that young people spend one‐third of their time at school, making them an ideal setting for promoting health and engendering preventative health behaviours from an early age [14, 15]. Developing physical and health literacy skills in schooling years has been identified as a global health promotion strategy that could significantly reduce the risk of morbidity for the future population, with the World Health Organization (WHO) and United Nations Educational, Scientific and Cultural Organization (UNESCO) launching a global initiative to make all schools ‘health‐promoting schools’ [15]. Health‐promoting schools create conditions that are conducive to health at all levels of the school through the implementation of policies, practices and targeted health curriculum, environment modification and connection with supports and local health networks [16]. Central to this are teachers who remain highly influential role models in young people to develop healthy routines and preventative behaviours [17]. However, research indicates that there are significant barriers for teachers in schools when attempting to increase young people's PA levels [18] and reduce ultraviolet radiation exposure [19] and improve sleep [20].

Supplementing existing initial teacher education programs to include targeted health promotion content to upskill preservice teachers before they enter schools has been reported to be a potentially feasible and sustainable strategy to improve the provision of knowledge and practices for preventative health behaviours in Australian and NZ schools [21, 22, 23]. In Victoria, researchers have trialled the ‘Transform Ed!’ program which is embedded into the initial teacher education degree and was designed to equip primary preservice teachers with the skills to be able to provide more meaningful PA experiences and outcomes. After the program, preservice teachers reported they were more willing, confident and competent to implement PA pedagogical strategies and perceived less barriers [24]. Other research with preservice teachers (*n* = 275) at one Australian University indicated significant gaps in ultraviolet radiation awareness and perceived knowledge, skills and confidence to teach sun safety [25]. After attending a brief university‐based intervention, preservice teachers (*n* = 161) indicated they felt the intervention increased their awareness of the dangers of overexposure to ultraviolet radiation, with many feeling more knowledgeable, skilled and confident to teach sun safety [26]. Preservice teachers supported a need for more consistent sun protection messaging across Australian schools with greater emphasis on ultraviolet radiation education and tailored curriculum implementation, rather than compliance management. The intervention was well‐received by preservice teachers and there was unanimous agreement among participants that all initial teacher education programs across Australia should include this targeted health promotion material so that all graduate teachers are properly equipped to protect young people from overexposure to ultraviolet radiation [22]. While research supports the value of sleep education for schoolteachers and young people [27], there is limited research evaluating the value of sleep education in initial teacher education for preservice teachers warranting further research.

While PA, sleep and sun safety have been identified as important modifiable health behaviours to prevent the onset of chronic disease and schools have been identified as an important setting for health promotion and primary prevention; little is known about how teacher education programs across Australia and NZ are preparing future teachers to deliver physical activity, sleep and sun safety (PASS) education in early childhood and school settings. Hence, this study investigated teacher educators' perceptions of existing early childhood, primary and secondary teacher education programs in Australia and NZ to obtain insights into how their programs are preparing future teachers to teach PASS education.

## 2 Methodology

### 2.1 Aims

This study aimed to:

1. Examine the estimated number of courses and amount of time being spent on PASS within existing initial teacher education programs in Australia and NZ.
2. Summarise Australian and NZ teacher educators' perceptions of graduates' knowledge, confidence and competence in delivering PASS content.
3. Explore differences according to country (Australia/NZ), teaching specialisation (Health and Physical Education/other), and discipline (early childhood/primary/secondary) in the number of courses and time spent teaching PASS content, as well as teacher educators' perceptions of graduates' knowledge, confident and competence.

### 2.2 Study Design and Participants

This study used an electronic cross‐sectional survey administered Nov 2023‐June 2024. Recruitment invitations were circulated via various social media platforms, relevant professional associations and emailed to leads of initial teacher education programs in Australia (*n* = 48) and NZ (*n* = 25). From here forth, entire initial teacher education programs or degrees will be referred to as ‘program’ and courses or units within the initial teacher education programs will be referred to as ‘course’. Invitations contained an online link and QR code to the project description and consent information page. Participants could also opt‐in to go in a prize draw to win a smartwatch. A total of (*n* = 187) consented to being involved in the study, however, (*n* = 6) were excluded from the study as they confirmed they were not employed at an institution that offered an initial teacher education program in Australia or NZ. A further (*n* = 83) were excluded due to incomplete data where they did not answer a minimum of five survey items. The final sample of (*n* = 98) was included in the analysis.

### 2.3 Survey

Participants completed the anonymous electronic Qualtrics survey [28] that was designed for the purposes of this study. The survey contained 30 questions (seven × demographic questions, 23 × attitudinal questions related to teacher education). To ensure that participants could remain anonymous, they were not required to provide the name of their institution, only their state/country, role, experience and teaching specialisation. For questions where participants had to estimate numerical values for time spent teaching content or the number of courses included in programs, a response slider was provided. For attitudinal questions, participants were provided with statements and a 7‐point scale (ranging from strongly disagree/disagree/don't know/agree/strongly agree category) as has been suggested as the most useful for accurately collecting attitudinal data [29].

### 2.4 Analysis

Survey data was downloaded from Qualtrics and imported into IBM SPSS software 29 (SPSS Inc. Chicago, IL) for quantitative analysis. Shapiro–Wilk tests were used for normality testing. Descriptive and frequency analysis was used to examine features of the data. For analysis of the teacher educators' attitudinal response data, variables were collapsed into three categories (disagree/don't know/agree). To examine differences between respondents from health and physical education backgrounds and those from other teaching areas, the teaching specialisation variable was collapsed into two groups (health and physical education/other). For non‐parametric ordinal data, independent‐samples Kruskal‐Wallis H tests with pairwise comparisons (*α* > 0.05) were used to explore group differences in the data based on country, state, teaching specialisation and discipline. Friedman ANOVA with Wilcoxon post hoc tests (*α* > 0.05) were used to explore differences in teacher educators' perceptions of PASS content and delivery in the initial teacher education programs.

## 3 Results

A final sample of (*n* = 98) teacher educators were included in the data analysis; 82% of were employed at Australian institutions with the remaining participants from NZ, which is representative of each country's populations (26 million and 5 million, respectively) [30]. Role in the institutions included: teacher/tutor (20%), course coordinator (46%), program coordinator (13%), discipline lead (7%) and Head/Dean of School (10%). Their years of teaching experience varied from 5 to 15 years and their specialisation was diverse and included: English, Maths, Health and Physical Education, Science, Humanities and Social Sciences, the Arts, Technologies and Languages among others (Table 1).

**TABLE 1:** Demographics of the sample (*n* = 98).

Category	Component	*n* (%)
Country	Australia	81 (82.7)
	New Zealand	17 (17.3)
Australian state^a^	QLD	28 (28.6)
	NSW	17 (17.3)
	VIC	8 (8.2)
	SA	3 (3.1)
	WA	25 (25.5)
Aotearoa NZ island	North	15 (88.2)
	South	2 (11.8)
Role in university	Tutor/teacher	20 (20.4)
	Course level coordinator	45 (45.9)
	Program coordinator	13 (13.3)
	Discipline lead	7 (7.11)
	Head/dean of school	10 (10.2)
	Missing	3 (3.1)
Experience in initial teacher education (years)	< 5 years	23 (23.5)
	6–10 years	26 (26.5)
	11–14 years	18 (18.4)
	> 15 years	31 (31.6)
Teaching specialisation	English	14 (14.3)
	Maths	12 (12.2)
	Health and physical education	19 (19.9)
	Science	5 (5.1)
	HASS	11 (11.2)
	The arts	3 (3.1)
	Technologies	5 (5.1)
	Languages	4 (4.1)
	Other	25 (25.5)
Discipline	Early childhood	13 (13.2)
	Primary	44 (44.9)
	Secondary	36 (36.8)
	Other	5 (5.1)

^a^ Nil responses from TAS, ACT, NT.

### 3.1 Number of Courses and Time Spent on PASS Content in Initial Teacher Education Programs

Considering entire initial teacher education programs at institutions, Friedman ANOVA with post hoc Wilcoxon signed rank tests indicated significant differences between PA, sleep and sun safety in terms of the number of courses which include PASS content, time spent in hours teaching PASS content, and time spent teaching preservice teachers how to plan and teach PASS content (*p* < 0.001) (Table 2). The number of courses within the entire initial teacher education programs that focus on PA (mean = 4.96, SD = 4.57), was significantly higher than for sun safety education (mean = 2.61, SD = 4.07) and sleep education (mean = 1.09, SD = 3.10). While only 2% of participants reported that programs at their institution include no courses which contain PA education, 20% of participants reported no courses included sleep education and 11% reported none that included sun safety education. Time in hours spent on PA education (mean = 26.09, SD = 25.69) across entire programs far outweighed time spent on sun safety education (mean = 8.48, SD = 18.77) and sleep education (mean = 5.79, SD = 15.21). When estimating the amount of time spent in hours educating preservice teachers on how to plan and effectively deliver education, PA again was significantly higher (mean = 27.09, SD = 28.02) than sun safety (mean = 5.95, SD = 14.29) and sleep (mean = 4.18, SD = 9.91). A total of 4% of participants reported their programs spent no time on educating teachers how to effectively plan and deliver lessons that focus on PA, whereas over 21% of participants reported that their entire programs spent no time at all teaching preservice teachers how to plan and effectively delivery sun safety and sleep content.

**TABLE 2:** Estimated number of courses and time spent focusing on PASS education in Australian and New Zealand initial teacher education programs.

	*N*	0 *n* (%)	1–2 *n* (%)	3–4 *n* (%)	≥ 5 *n* (%)	Unsure^a^ *n* (%)	Mean	SD	*p* ^b^
Number of courses in program that include									
Physical activity education	83	2 (2.0)	32 (32.7)	20 (20.4)	29 (30)	15 (15.3)	4.96	4.57	
Sun safety education	69	11 (11.2)	38 (38.8)	12 (12.2)	8 (8.2)	29 (29.6)	2.61	4.07	< 0.001
Sleep education	67	20 (20.4)	34 (34.7)	6 (6.1)	7 (7.1)	31 (31.6)	1.90	3.10	
Time (hours) spent in program on									
Physical activity education	76	2 (2.0)	7 (7.2)	5 (5.1)	62 (63.3)	22 (22.4)	26.09	25.69	
Sun safety education	67	13 (13.3)	22 (22.4)	7 (7.1)	25 (25.5)	31 (31.6)	8.48	18.77	< 0.001
Sleep education	66	16 (16.3)	25 (25.5)	9 (9.2)	16 (16.3)	32 (32.7)	5.79	15.21	
Time (hours) spent in program on planning and effectively delivering									
Physical activity lessons	79	4 (4.1)	5 (5.1)	7 (7.2)	63 (64.3)	19 (19.4)	27.09	28.20	
Sun safety lessons	66	21 (21.4)	21 (21.4)	8 (8.2)	16 (16.2)	32 (32.7)	5.95	14.29	< 0.001
Sleep lessons	62	22 (22.4)	22 (22.4)	6 (6.1)	12 (12.2)	36 (36.7)	4.18	9.91	

^a^ Participants ticked “I don't know” instead of providing an estimate.

^b^ Friedman tests used to examine difference between time spent on physical activity, sun safety and sleep content.

### 3.2 Graduates' Knowledge, Confidence and Competence to Teach PASS Content

While most teacher educators believed that their graduates received sufficient training on PA education (65.5%) and the associated health benefits (78.6%), the burden associated with inactivity (72.4%), and understanding of current guidelines (58%); these numbers were far lower for sun safety and sleep (*p* < 0.001) (Table 3). In addition, > 40% of participants reported they did not know if their graduates received sufficient sun safety or sleep training, nor if they understood the associated benefits (35% and 37%, respectively), risks (34% and 34%, respectively) and guidelines (38% and 48%, respectively). Most participants also indicated that they believed their graduates were confident (71.4%) and competent (70.4%) to plan and teach lessons that focused on PA education. However, this was significantly different to their perceptions of graduates' confidence and competence to teach lessons focused on sun safety (*p* < 0.001) and sleep (*p* < 0.001) content. In terms of sun safety, only 41.8% felt their graduates were confident and 45.9% felt they were competent. When asked about sleep, most of the sample (> 70%) reported they did not agree or did not know if their graduates were confident to plan and teach lessons that focus on sleep education.

**TABLE 3:** Teacher educator's perceptions of graduates' knowledge, confidence and competence to teach physical activity, sun safety and sleep (*n* = 98).

	Disagree *n* (%)	Don't know *n* (%)	Agree *n* (%)	Missing *n* (%)	Mean	SD	^*a*^ *p*
Graduates receive sufficient training that focuses on							
Physical activity	17 (17.3)	17 (17.3)	64 (65.3)	0 (0)	2.47	0.78	
Sun safety	21 (21.6)	43 (43.9)	33 (33.7)	1 (1.0)	2.12	0.74	< 0.001
Sleep	32 (32.7)	40 (40.8)	24 (24.5)	2 (2.0)	1.91	0.76	
Graduates understand the health benefits associated with							
Physical activity	9 (9.2)	12 (12.2)	77 (78.6)	0 (0)	2.7	0.61	
Sun safety	14 (14.3)	34 (34.7)	49 (50)	1 (1.0)	2.36	0.73	< 0.001
Sleep	19 (19.4)	36 (36.7)	41 (41.8)	2 (2.0)	2.23	0.76	
Graduates understand the health risks associated with							
Physical inactivity	8 (8.2)	19 (19.4)	71 (72.4)	0 (0)	2.63	0.63	
Lack of sun safety	14 (14.3)	33 (33.7)	50 (51.0)	1 (1.0)	2.37	0.73	< 0.001
Poor sleep	21 (21.4)	33 (33.7)	42 (42.9)	2 (2.0)	2.21	0.78	
Graduates know and understand current national guidelines for							
Physical activity	12 (12.2)	29 (29.6)	57 (58.2)	0 (0)	2.47	0.71	
Sun safety	18 (18.4)	37 (37.8)	42 (42.9)	1 (1.0)	1.97	0.72	< 0.001
Sleep	26 (26.5)	47 (48.0)	23 (23.5)	2 (2.0)	2.25	0.75	
Graduates are confident to plan and teach lessons focuses on							
Physical activity content	10 (10.2)	18 (18.4)	70 (71.4)	0 (0)	2.63	0.66	
Sun safety content	19 (19.4)	37 (37.8)	41 (41.8)	1 (1.0)	2.23	0.76	< 0.001
Sleep content	30 (30.6)	44 (44.9)	22 (22.4)	2 (2.0)	1.91	0.74	
Graduates are competent to plan and teach lessons focuses on							
Physical activity content	10 (10.2)	19 (19.4)	69 (70.4)	0 (0)	2.61	0.67	
Sun safety content	17 (17.3)	35 (35.7)	45 (45.9)	1 (1.0)	2.29	0.75	< 0.001
Sleep content	31 (31.6)	41 (41.8)	24 (24.5)	2 (2.0)	1.92	0.76	

^a^ Difference between physical activity, sun safety and sleep (alpha: 0.05).

### 3.3 Group Differences Based on Country, Specialisation and Discipline

#### 3.3.1 Country

There were no significant differences between Australia and NZ other than the number of courses offered in the program that focused on sleep (*H* = 4.47; *p =* 0.035); and how many hours were spent on PA content (*H* = 3.87; *p =* 0.049), with Australia having higher means in both than NZ. There was no difference between states within Australia in terms of time spent on PASS content or perceptions of their graduates' knowledge, confidence or competence (*p* > 0.05).

#### 3.3.2 Teaching Specialisation

Teacher educators with health and physical education specialisation had more favourable perspectives when compared to those from other learning areas about their own graduates' knowledge of PA benefits (*p =* 0.002), risks (*p <* 0.001) and guidelines (*p =* 0.008) and ability to plan and teach lessons focusing on this content (*p =* 0.008). However, this was not the case for sun safety (*p* > 0.05) or sleep (*p* > 0.05) and there were no other differences in perspectives based on time spent on PASS content or graduates' knowledge, confidence and competence to teach sun safety or sleep content (*p* > 0.05).

#### 3.3.3 Discipline

There was no overall difference in the number of courses offered that included PASS content between early childhood, primary and secondary disciplines for PA (*p* > 0.05) or sun safety (*p* > 0.05). However, early childhood teacher educators reported higher number of courses that included sleep content (*p =* 0.02) and higher number of hours allocated to both sleep (*p* < 0.01) and sun safety (*p* < 0.01) education. There was no difference across disciplines in terms of graduates' knowledge of PA benefits, risks and guidelines (*p* > 0.05). However, teacher educators from the early childhood discipline perceived their graduates to have higher knowledge of sun safety and sleep benefits, risks and guidelines than both primary (*p* < 0.01) and secondary (*p* = 0.02) programs.

## 4 Discussion

To the authors’ knowledge, this study was the first study to investigate initial teacher educators' perceptions of their existing programs in relation to the amount of content and delivery of PASS education in Australia and NZ. Findings indicate there were minimal differences between Australian and NZ programs, other than the number of courses that focus on sleep and how many hours were spent on PA content. There was also no difference between states within Australia in terms of time spent on PASS content or perceptions of their graduates' knowledge, confidence or competence. However, there was significant variance in what is being offered in terms of the amount of content, curriculum focus and perceived teacher preparedness to teach PASS‐related content.

Based on the findings, most initial teacher education programs in Australia and NZ spent very little (< 3 h to no time) in the entire initial teacher education program for each of the PASS areas. With between 10%–32% of participants reporting that they do not feel that their graduates are confident and competent to teach lessons that focus on PASS content, it is indicated that the current amount of content within existing programs in Australia and NZ is insufficient. In both Australia and NZ, PA had the highest coverage in programs, followed by sun safety and sleep. Teacher educators also perceived their graduates had the least knowledge of benefits, risks and guidelines and perceived confidence and competence to plan and deliver sleep‐related content. This may be due to the fact that the promotion of PA and sun safety and related policies has been a focus in schools for the last couple of decades [31, 32], whereas sleep is relatively new on the public health agenda [33] with Australia only including sleep in the children and young people's 24‐h movement guidelines in 2018 [34]. These findings could speak to the need for tertiary institutions to examine and modify their existing initial teacher education programs to include more comprehensive sleep education content. Of concern, a large proportion of the sample (ranging from 15%–36% depending on the question) reported they “did not know” how many courses or hours were allocated to PASS education in their program. However, as a large proportion of the sample (approximately 65%) were not program coordinators, discipline leads or heads of school and based on their role in the institution may only have knowledge of the courses they teach and not deep knowledge of the whole program. Regardless, these findings could highlight a potential need for initial teacher education providers to cross reference all course content via regular review to ensure that all teachers and educators who are teaching these programs are aware of what is covered in the broader program and identify existing gaps.

Examination of group differences between perceptions of health and physical education teacher educators and those in other specialisations revealed no significant difference in their estimates of the amount of PA content in their programs. This was an unexpected finding as those with a health and physical education specialisation had more favourable perceptions of their preservice teachers' preparedness to plan and deliver PA‐related lessons and their knowledge of PA benefits, risks and guidelines. However, there were no differences in perspectives on sun safety or sleep when educators were grouped by specialisation. Our findings also indicated no overall differences between early childhood, primary and secondary programs in the number of courses that include PA or sun safety education, but there was for sleep with early childhood and primary having higher means than secondary. In addition, early childhood educators reported a higher number of allocated hours for both sleep and sun safety and were also more favourable in their reporting about their graduates' knowledge of the risks, guidelines and benefits of these health behaviours. In Australia, this could be a result of the fact that early childhood settings are regulated by national law in which the Australian Children's Education & Care Quality Authority (ACECQA) sets a national quality standard for children's care with particular standards for health, well‐being and safety [35]. Accreditation requirements for early childhood initial teacher education programs may reflect these requirements. While primary and secondary have similar accreditation requirements and professional teaching standards set by the Australian Institute for Teaching and School Leadership (AITSL) [36], there is potentially less prescriptive guidance in terms of some of these health behaviours leading to institutions potentially placing larger focuses on other content more closely aligned to the wording in the teaching standards.

For national curriculum used by Australian and NZ school teachers set by the Australian Curriculum, Assessment and Reporting Authority (ACARA) [37] and the Ministry of Education (MoE) in NZ [38], PA and personal safety (including sun) are most explicitly mentioned in the health and physical education learning area. In Australia, PA is a requirement at every year level; however, sun safety is a ‘suggested topic’ and not a requirement at any year level. Cancer Council Australia and Cancer Society New Zealand's SunSmart Schools and Early Childhood Program require registered members to include sun protection in the curriculum for all year levels [31, 39]. However, not all schools or early childhood services are registered members and sun education and protection strategies remain inconsistent across schools [26] with school communities requiring additional support and engagement to effectively implement programs [40]. Sleep as a topic is not explicitly mentioned by ACARA or MoE in either health or physical education curriculum [37, 38]. This limits guidance for schools for when and where sleep education should (or could), be taught in a school program and potentially could lead to sleep education being omitted from school curriculum programs.

Health and physical education as a curriculum learning area has historically had the acquisition of movement skills at its core [37], which is likely the reason for schools prioritising PA education over health behaviours as it is more prescriptive. As the ITE program curriculum is modified based on sociocultural changes in schools and national curriculum, this may be a reason for the prioritisation of PA over other health behaviours in ITE programs in Australia and NZ. With contemporary advancements in technology, media and their relationship with health behaviours and risks, all school curriculum learning areas have gone through significant reform [41, 42] and now include education focusing on young peoples' personal and social capability and their relationships with the outside world [37, 38]. We therefore argue that knowledge of current guidelines, risks and benefits related to health behaviours is particularly important for *all* preservice teachers regardless of where they study, the program, discipline or specialisation. However, findings of this study indicate that different institutions across Australia are offering different amounts of PASS content within initial teacher education programs and also how much time is spent teaching preservice teachers to plan and teach PASS‐related content. This gap may lead to graduate teachers entering schools with little understanding of some, or all of health behaviours, associated risks and broader health implications for young people which is of significant concern, as teachers have been shown to be influential health promotors in both primary and secondary schools [14].

Our study provides interesting insights into existing initial teacher educator programs in Australia and NZ. However, there are some limitations that should be noted. First, as the overarching aim of the study was to examine PASS education in existing initial teacher education programs, rather than compare institutional offerings, participant institution data was not collected. While it allowed anonymity, it is a noted limitation of this study as it did not allow direct comparison of what individual institutions are offering within their initial teacher education program. Furthermore, it did not allow us to determine if multiple staff from one institution have completed the survey, leading to potential overlap of data. Second, the study included a relatively small sample of teacher educators (*n* = 98), which may limit the generalisability of findings. Third, due to the limited scope of this small study, we were only able to focus on three behaviours which are known to reduce risk of developing cancer or chronic disease in later life [43, 44, 45]. However, it is important to note that there are other modifiable health behaviours that reduce cancer and chronic disease risk including nutrition, smoking, alcohol and other substance use and mental health [46] are also important and warrant further research.

## 5 Conclusion

This novel study was the first to investigate initial teacher educators' perceptions of their existing early childhood, primary and secondary initial teacher education programs in relation to the amount of content and delivery of PASS education in Australia and NZ. The findings revealed there was significant variance in what is being offered in terms of the amount of content, curriculum focus and perceived teacher preparedness to teach PASS‐related content. Consistently, time in hours spent on PA education across the entire initial teacher education programs far outweighed time spent on sun safety education and sleep education. Many teacher educators revealed their programs had little or no sleep or sun safety content and did not agree their graduates were confident to plan and deliver sun safety and sleep content. As *all* teachers need to have good knowledge of lifestyle‐related health behaviours to support young people's health effectively and efficiently, initial teacher education providers are encouraged to review their existing programs to explore how they can best prepare their graduate teachers to be health promotors including resourcing, planning, reviewing and modifying existing programs. Supplementing existing initial teacher education programs to include greater PASS education to upskill preservice teachers before they enter schools may provide a feasible and sustainable health promotion and cancer prevention strategy in Australian and NZ schools, however further research is needed to further examine institutional differences and the most efficient and effective ways to integrate lifestyle behaviour‐related content into existing initial teacher education programs. There is a need to examine, refine and modify public health content in Australian and NZ teacher education programs to assist with delivering consistent public health messages in schools via teachers. Further research investigating barriers and facilitators for modifying initial teacher education programs is needed.

## Author Contributions

All authors are responsible for reported research and have participated in the concept and design and/or data collection and/or analysis and interpretation of data, drafting or revising, and have approved this manuscript as submitted.

## Ethics Statement

The study was approved by the relevant university Human Research Ethics Committees in Australia and New Zealand (Approval number: A231855).

## Conflicts of Interest

The authors declare no conflicts of interest.

## Data Availability

The data that support the findings of this study are available on request from the corresponding author. The data are not publicly available due to privacy or ethical restrictions.
